# Great Britain and the United States

**Published:** 1917-04-28

**Authors:** 


### The Hospital

The Workers' Newspaper of Administrative Medicine and Institutional Life, Administration, National Insurance and Health.

No. 1612, Vol. LXII. SATURDAY, APRIL 28, 1917. PRICE ONE PENNY

### GREAT BRITAIN AND THE UNITED STATES.

Many momentous and wonderful things have happened since the Kaiser and Germany set out to destroy the freedom of the nations by the exercise of organised militarism and brute force, divorced from every restraint imposed by humanity, chivalry, justice, morality, and righteousness. German Kultur in practice has proved itself to mean, on the part of the All-Highest, his sons, and his naval and military leaders, a continuous series of the most brutal and appalling crimes. With the approval of every class of the German population, both men and women, Germans have exhibited to the world deeds and acts which have been so vile, unsportsmanlike, and degrading as to surpass anything done previously by human beings or members of the animal kingdom since the world was made. It is not surprising that the result has been to unite practically the whole of civilised humanity in the assured conviction that a nation so degenerate, and so lost to all human feeling, must be broken, and deprived of the power of ever again attempting to intimidate and enslave the world.

The most momentous, wonderful, and glorious result of the great war has been the spontaneous uniting of the Anglo-Saxon race. A united Anglo-Saxon race, the inter-co-operation and unity of all races speaking the English tongue in every part of the world, should mean the enforcement of Christian principles, freedom for everybody, and the best things in life which the wisest and most democratic Governments can secure for all classes of their people. The mother and her children in St. Paul's Cathedral on the 20th inst. were united in a solemn service to Almighty God on the occasion of the entry of the United States of America into the great war for freedom. This rejoicing of the mother and her children's dedication should mean a much closer and more intimate co-operation than ever before. Britain and the United States have learnt, and are learning, and must continue to learn much from each other. In the last fifty years the progressive improvements and developments which both countries owe to science, the interchange of knowledge, the widening of business enterprise, the invention and perfecting of machinery, and the advance which has taken place in engineering, traction, railway, and mechanical resources, have been immense. We look with assured certainty to the extension and growth of all these important aids to human progress in the near future.

We have had the good fortune to keep in constant touch with Canada and the United States for upwards of thirty years, and are personally as familiar with much of the life and progress of our brothers across the Atlantic as we are with that of the people of our own country. It was our duty to visit America last autumn, and to be resident there during the late Presidential election. That election was remarkable because no one could or would forecast the result, owing to an almost equal division of public feeling as to the merits of each candidate for the Presidency. Before the election, as the American Hospital Conference showed, the German element was strong enough to make itself felt, and so strong was the desire to keep out of the war that *The Modern Hospital* rebuked an Englishman for mentioning the war and alluding to the hospital and ambulance experience of Great Britain, its difficulties and lessons. Yet within six months the United States has spontaneously entered the war and joined hands with the Mother Country.

We have felt, therefore, that to-day we might helpfully exhibit to our readers and the British people the close connection which exists between the highest standards of Hospital administration, by comparing the best types to be found to-day in the United States and the United Kingdom. Even so restricted, the field is wide enough to cover many branches of work of momentous importance to the human race.

